# Fatal Influenza B–MRSA Coinfection in a Healthy Adolescent: Necrotizing Pneumonia, Cytokine Storm, and Multi-Organ Failure

**DOI:** 10.3390/children12060766

**Published:** 2025-06-13

**Authors:** Irina Profir, Cristina-Mihaela Popescu, Aurel Nechita

**Affiliations:** 1 Faculty of Medicine and Pharmacy, “Dunărea de Jos” University of Galați, 800010 Galați, Romania; irina.profir@ugal.ro (I.P.); aurel.nechita@ugal.ro (A.N.); 2 “Sf. Ioan” Clinical Emergency Children’s Hospital, 800487 Galați, Romania

**Keywords:** influenza B virus, acute respiratory distress syndrome, respiratory tract infections, methicillin-resistant staphylococcus aureus, cytokine storm, rhabdomyolysis, necrotizing pneumonia, vaccination

## Abstract

Background: Influenza B usually causes mild illness in children. Severe and fatal cases can occur when complicated by secondary *Staphylococcus aureus* (*S. aureus)* pneumonia, including community-acquired methicillin-resistant *Staphylococcus aureus* (MRSA). We present a rare, rapidly progressive fatal case in an adolescent with no known medical history to highlight diagnostic and therapeutic pitfalls. Case Presentation: A 16-year-old boy with no known underlying conditions (unvaccinated for influenza) presented critically ill at “Sf. Ioan” Clinical Emergency Pediatric Hospital in Galați after one week of high fever and cough. He was in respiratory failure with septic shock, requiring immediate intubation and vasopressors. Chest X-ray (CXR) showed diffuse bilateral infiltrates (acute respiratory distress syndrome, ARDS). Initial laboratory tests revealed leukopenia, severe thrombocytopenia, disseminated intravascular coagulation (DIC), rhabdomyolysis, and acute kidney injury (AKI). Reverse transcription polymerase chain reaction (RT-PCR) confirmed influenza B, and blood cultures grew MRSA. Despite maximal intensive care, including mechanical ventilation, antibiotics (escalated for MRSA), antiviral therapy, and cytokine hemoadsorption therapy, the patient developed refractory multi-organ failure and died on hospital day 6. Autopsy revealed bilateral necrotizing pneumonia (NP) without radiographic cavitation, underscoring the diagnostic challenge. Discussion: The initial chest radiography showed diffuse bilateral pulmonary infiltrates, predominantly in the lower zones, with an ill-defined, patchy, and confluent appearance. Such appearance, in our case, was more suggestive of rapid progressive NP caused by MRSA rather than the typical pneumococcal one. This is one of the few reported cases of influenza B–MRSA coinfection with fulminant rhabdomyolysis and autopsy-confirmed necrosis. Our fulminant case illustrates the synergistic virulence of influenza and MRSA. Toxin-producing MRSA strains can cause NP and a “cytokine storm,” causing capillary leak, ARDS, shock, and DIC. Once multi-organ failure ensues, the prognosis is grim despite aggressive care. The absence of early radiographic necrosis and delayed anti-MRSA therapy (initiated after culture results) likely contributed to the poor outcome. Conclusions: Influenza B–MRSA co-infection, though rare, demands urgent empiric anti-MRSA therapy in severe influenza cases with leukopenia or shock, even without radiographic necrosis. This fatal outcome underscores the dual imperative of influenza vaccination and early, aggressive dual-pathogen targeting in high-risk presentations.

## 1. Introduction

Influenza viruses are a significant cause of respiratory infections worldwide. Although influenza B typically causes mild to moderate illness in healthy children, it can lead to severe disease and even death [1]. Pediatric patients often experience more pronounced symptoms than adults, and influenza B outbreaks have been associated with higher morbidity and hospitalization rates in children [1]. Viral pneumonia and secondary bacterial infection are well-recognized complications of influenza. *S. aureus* is the most frequently reported pathogen, followed by *Streptococcus pneumoniae*, *Streptococcus pyogenes*, *Haemophilus influenzae*, and, less commonly, *Neisseria meningitidis* and *Moraxella catarrhalis* [2]. *S. aureus*, especially community-associated, toxin-producing strains, is notorious for causing fulminant NP after influenza [3,4]. Such viral–bacterial co-infections can lead to rapid clinical deterioration in less than 48 h [5]. Co-infection with influenza virus and *S. aureus* potentiates an exaggerated immune response, resulting in excessive cytokine release, endothelial injury, and progression to ARDS, shock, and coagulopathy [3,4]. In this context, NP is a rare but devastating event, with reported mortality rates as high as 40–45% [3,6].

We describe a fatal case of influenza B in an adolescent male with no known medical history, complicated by secondary community-acquired MRSA, NP, ARDS, refractory septic shock, rhabdomyolysis, AKI, DIC, and acute liver failure. This case highlights the preventive role of influenza immunization and the importance of maintaining high clinical vigilance and aggressive management of bacterial co-infections in severe influenza, even in young, immunocompetent patients [3,6]. Similar fulminant influenza B–MRSA co-infections have rarely been reported in the literature [3].

## 2. Case Presentation

### 2.1. History

A 16-year-and-5-month-old boy, with no known underlying conditions and no history of influenza vaccination, developed an abrupt onset of high fever (≥39.5 °C) and dry cough about one week before admission. He was initially evaluated by a general practitioner and treated empirically with antipyretics and oral azithromycin for a presumed community-acquired pneumonia. Despite this outpatient therapy, his fever persisted, and his cough worsened. Two days before hospital presentation, he developed progressively severe dyspnea and profound fatigue. There was no known exposure to sick contacts and no recent travel history. His family sought emergency medical care when he became markedly short of breath and lethargic.

### 2.2. Examination on Admission

Upon arrival at the emergency department of “Sf. Ioan” Clinical Emergency Pediatric Hospital in Galați, the patient was in extremis. The patient presented somnolent and pale, with signs of severe respiratory distress. He exhibited perioral cyanosis (SpO_2_ <70% on high-flow oxygen) and hypotension (blood pressure ~80/50 mmHg) with an inappropriately low heart rate (~50 bpm) relative to his shock, consistent with cold (hypodynamic) septic shock. Capillary refill was delayed (>4 s), and his extremities were cool. Heart sounds were rapid but faint; lung auscultation revealed diffuse bilateral crackles. The patient’s mental status was depressed (Glasgow Coma Scale score 10 points) due to hypoxemia and poor perfusion. In light of the severe acute respiratory failure and shock, the decision was made for immediate aggressive supportive therapy. This consisted of mechanical ventilation, fluid resuscitation, vasoactive support with inotropes, broad-spectrum antibiotics, corticosteroids, blood product transfusions, renal replacement therapy, cytokine hemoadsorption, and consideration for extracorporeal membrane oxygenation (ECMO), which was ultimately not initiated due to rapid clinical deterioration and institutional limitations.

### 2.3. Case Evolution

Initial laboratory studies showed severe leukopenia (white blood cell 1.7 × 10^3^/µL) and thrombocytopenia (platelets 56 × 10^3^/µL). Coagulation tests indicated a consumptive coagulopathy (International Normalized Ratio ~1.6, prolonged PT). Inflammatory markers were dramatically elevated (C-reactive protein 25.3 mg/dL [<0.5], procalcitonin 675 ng/mL [<0.5], ferritin 980 ng/mL, IL-6 > 5000 pg/mL), consistent with a severe hyperinflammatory response. Early rhabdomyolysis was present (CK 661 U/L), and AKI was evident (serum creatinine 1.5 mg/dL; baseline ~0.8 mg/dL). The pediatric Sequential Organ Failure Assessment (pSOFA) score was 11 at admission, reflecting multi-organ involvement. Blood lactate was 6.1 mmol/L (lab data are provided in Table 1, Table 2 and Table 3; Day 1–Day 6-hospitalization days).

**Figure 1:**
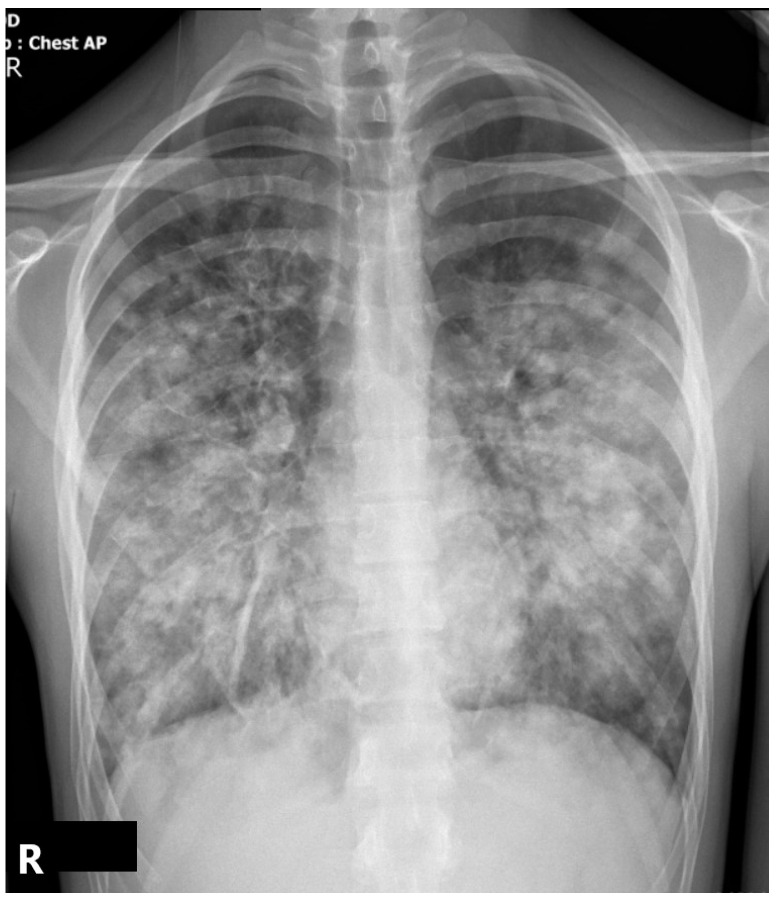
Anteroposterior CXR showing diffuse infiltrates consistent with early ARDS. No cavitation is noted (R-right side of the patient).

The initial chest X-ray demonstrated diffuse bilateral pulmonary infiltrates, particularly in the lower lobes, with a patchy, ill-defined, and confluent pattern. Considering the clinical context, these findings were highly suggestive of early NP, most consistent with *S. aureus* etiology. Typical community-acquired pneumococcal pneumonia has a segmental or lobar consolidation aspect (Figure 1).

**Figure 2:**
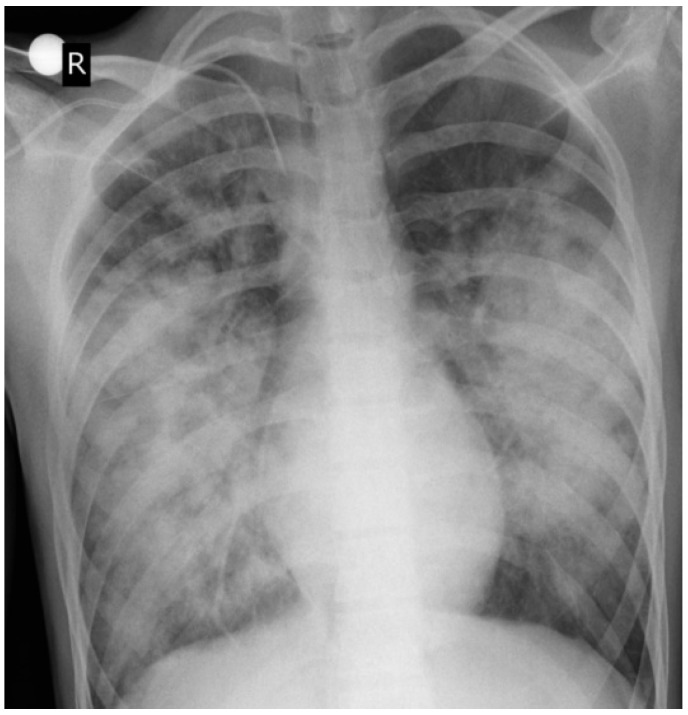
Anteroposterior CXR. Eight hours after the CXR in Figure 1. Rapid progression of the bilateral opacities (R-right side of the patient).

In the first hours of intensive care, a follow-up CXR taken the same day showed rapid progression of the opacities (Figure 2).

Abdominal ultrasound revealed a moderate volume of free fluid in the pelvis and between intestinal loops (ascites up to ~5 cm depth), consistent with third spacing due to capillary leak.

Microbiological tests were performed, including blood cultures, nasopharyngeal swab RT-PCR for respiratory viruses (including influenza A and B), multiplex PCR for atypical pathogens, and culture of tracheal aspirate. The nasopharyngeal swab was positive for influenza B virus by RT-PCR (RT-PCR Ct value and lineage determination are not currently performed in our institution’s laboratory).

The patient was admitted to the pediatric intensive care unit (PICU) and ventilated with pressure-regulated volume control (tidal volume ~6 mL/kg, positive end-expiratory pressure (PEEP) 10–15 cm H_2_O, plateau pressure <30 cm H_2_O). He met the criteria for severe ARDS (PaO_2_/FiO_2_ <100) [6]. Sedation and neuromuscular blockade were used to facilitate ventilator synchrony, achieved with midazolam, fentanyl, and propofol, while paralysis was maintained with atracurium. This approach aimed to reduce patient–ventilator dyssynchrony, minimize oxygen consumption, and enable lung-protective ventilation in the setting of severe ARDS, as recommended by pediatric critical care guidelines [7].

Hemodynamic support included a 60 mL/kg crystalloid bolus followed by norepinephrine infusion (0.1 µg/kg/min) for hypotension, with dopamine (5 µg/kg/min) added as myocardial dysfunction became apparent.

Empiric broad-spectrum antibiotics (IV meropenem-40 mg/kg every 8 h and vancomycin-15 mg/kg every 6 h) were initiated within the first hour of admission. Oseltamivir was started (75 mg twice daily) via nasogastric tube upon influenza B confirmation (Day 1), though this was already Day 8 of illness. High-dose methylprednisolone (2 mg/kg/day) was also started on Day 1 due to severe hyperinflammation.

On Day 2, the patient was placed in a prone position (6–16 h/day) for refractory hypoxemia. Vancomycin was switched to linezolid (10 mg/kg every 8 h) for better lung penetration and renal toxicity mitigation. Concurrently, creatinine rose from 1.5 to 2.3 mg/dL, CK reached 35,841 U/L, and IL-6 remained >8365 pg/mL. Continuous renal replacement therapy (CRRT) was initiated using a continuous veno-venous hemodiafiltration (CVVHDF) protocol with regional citrate anticoagulation. It was continued all throughout his hospitalization. A cytokine hemoadsorption cartridge was integrated into the CRRT circuit and applied for 3 consecutive days, with cartridges replaced every 24 h, in an effort to reduce systemic inflammation and support hemodynamic stabilization. Intravenous immunoglobulin (IVIG) (1 g/kg) was administered. Coagulopathy progressed with DIC, requiring transfusions of platelets and plasma.

On Day 3, CRP peaked at 37.8 mg/dL, CK rose to 56,422 U/L, and blood cultures flagged MRSA. PVL gene testing was not available at our institution; however, the clinical presentation was strongly suggestive of a PVL-producing strain of MRSA. Moxifloxacin was added (10 mg/kg once daily). Diagnosis: influenza B pneumonia with community-acquired MRSA sepsis, leading to ARDS, septic shock, DIC, rhabdomyolysis, and organ failure.

Despite full ICU support, ARDS remained severe. PaO_2_/FiO_2_ <70 persisted despite high PEEP, prone positioning, and protective ventilation. Urine output dropped to anuria. CRRT helped manage fluid overload (80–100 mL/h ultrafiltration).

**Figure 3:**
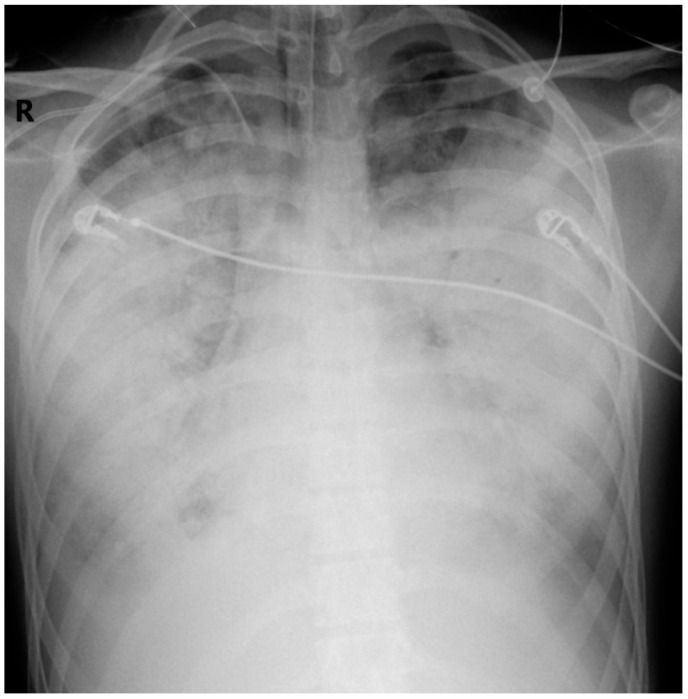
Anteroposterior CXR. Hospitalization Day 4. Further progression of the bilateral opacities (R-right side of the patient).

On Day 4, CK rose to 95,953 U/L; anasarca and pleural effusions (ultrasound confirmed) worsened respiratory function (Figure 3). Maximum doses of norepinephrine (titrated up to 1 µg/kg/min) and dopamine (titrated up to 20 µg/kg/min) were required. Lactate >6 mmol/L and LDH 3494 U/L signaled refractory shock.

By Day 5, the patient developed sinus tachycardia due to worsening hypoxemia and acidosis. PaO_2_/FiO_2_ <100 initially, worsening to <70 by Day 5, indicating critical hypoxemia. This was accompanied by progressive thrombocytopenia (lowest recorded value 13,900/mm^3^), DIC, and AKI (creatinine >2.0 mg/dL from Day 2), likely worsened by rhabdomyolysis. He remained deeply sedated (GCS score 3 points). Despite increasing doses of norepinephrine (up to 1 µg/kg/min) and dopamine (up to 20 µg/kg/min), the patient remained hypotensive with signs of refractory shock and progressive multiorgan failure.

Supportive care included ulcer prophylaxis (IV pantoprazole 1 mg/kg once daily) and stress-dose methylprednisolone (2 mg/kg/day). Parenteral nutrition (PN) was initiated with a standard three-in-one PN formulation administered via central venous access, including dextrose (5–10%), amino acids (2 g/kg/day), and lipid emulsion (1 g/kg/day), along with daily-adjusted electrolytes, vitamins, and trace elements. Deep venous thrombosis (DVT) prophylaxis was initiated using very low-dose unfractionated heparin (<10 units/kg/hour IV), tailored to minimize bleeding risk while maintaining vascular access and reducing thrombotic complications. Antifungal therapy was withheld due to a lack of evidence of fungal infection and short ICU duration.

The progression of organ dysfunction was reflected by a rising pSOFA score, which increased from 11 on admission to 15 by Day 5.

Day 6: AST spiked to 10,586 U/L, ALT to 2569 U/L, and CK to 104,280 U/L. The patient arrested (SpO_2_ 50–60% on 100% FiO_2_); resuscitation was unsuccessful after 45 min.

This daily progression highlights the rapid, refractory trajectory of fulminant MRSA pneumonia complicating influenza B, culminating in multi-organ failure and death.

The family gave their consent for the autopsy. It revealed bilateral acute NP, generalized fibrinous peritonitis, bilateral fibrinous pleuritis, and serous effusions, along with diffuse purpuric skin lesions and cerebral edema. Microscopically, findings included suspected acute myocarditis, ischemic hepatitis and tubular epithelial cell necrosis, moderate visceral congestion, and bilateral adrenal hemorrhage (likely related to shock/DIC).

All findings confirmed the clinical picture of multi-organ failure due to overwhelming influenza and MRSA infection. The cause of death was determined to be respiratory failure from MRSA NP complicating influenza B, with secondary multi-organ dysfunction.

## 3. Discussion

### 3.1. General Considerations

This case highlights the fulminant progression of influenza B in a previously well adolescent, complicated by community-acquired MRSA NP and multi-organ failure. Although often viewed as less severe than influenza A, influenza B can cause critical illness in children, with similar PICU outcomes [1,8]. The Romanian National Institute for Public Health did not upload the data for the latest flu season. However, the information available for the previous one shows that 5% of all influenza laboratory-confirmed cases were type B [9].

Viral epithelial damage impairs host defenses, facilitating bacterial superinfection, most critically with toxin-producing strains of *S. aureus*, particularly Panton–Valentine leukocidin (PVL)-positive MRSA [3,10]. NP, a rare but fatal complication, is more likely in previously healthy children with concurrent influenza, leukopenia, and lack of pneumococcal vaccination [2,11,12].

Although PVL-producing strains are infrequent in Europe (1–5%), they are disproportionately associated with severe, fulminant infections characterized by leukopenia, hemoptysis, and shock [2,9,11]. Radiographs may show patchy bilateral infiltrates, but early signs of necrosis are often subtle. In high-risk presentations, PVL-positive *S. aureus* should be suspected. The diagnostic workup includes blood cultures, respiratory PCR, and, if feasible, bronchoalveolar lavage. PVL gene confirmation is rarely available in real-time [12].

The differential diagnosis includes meningococcemia, viral myocarditis, toxic shock syndrome, and atypical pneumonias [13]. In this case, meningococcemia was ruled out via negative cultures and absent purpura, and toxic shock was not supported by mucocutaneous or gastrointestinal features. Myocarditis was considered, but there were no signs of isolated cardiac dysfunction or rhythm disturbance. The clinical picture was dominated by respiratory failure and septic shock rather than primary cardiac compromise. MRSA was confirmed by culture, with the clinical course consistent with NP.

Given these overlapping features, early recognition of post-influenza MRSA pneumonia remains difficult. Both viral pneumonitis and bacterial co-infection may present with leukopenia and diffuse infiltrates [14]. Although PVL testing was unavailable, leukopenia and rapid deterioration raised clinical suspicion for toxin-mediated MRSA [15].

### 3.2. Acute Respiratory Distress Syndrome (ARDS)

The patient met ARDS criteria at presentation, with bilateral infiltrates, severe hypoxemia, and no signs of cardiogenic edema [16]. In influenza, ARDS arises from both viral cytopathy and a heightened inflammatory response [17]. In co-infections with PVL-positive MRSA, necrotic lung injury, neutrophilic infiltration, and cytokine release lead to diffuse alveolar damage and surfactant loss [3,18,19].

Despite standard management, including lung-protective ventilation, proning, and neuromuscular blockade [20], hypoxemia remained refractory, reflecting severe lung damage. MODS is the primary cause of death in pediatric ARDS, contributing to around 50% of cases [21].

ECMO was unavailable; even where accessible, outcomes in NP are uncertain [22]. Although ECMO is a potential intervention in refractory pediatric ARDS, it was not available at our center. Furthermore, rapid hemodynamic deterioration, multi-organ failure, and refractory shock precluded safe transfer or candidacy for ECMO elsewhere. According to current ECMO guidelines, multi-organ dysfunction, refractory shock, and severe hepatic injury are recognized as relative or absolute contraindications to ECMO in pediatric patients with ARDS, particularly when the likelihood of reversibility is low or the risk of anticoagulation is prohibitive [13]. These factors, combined with institutional limitations, guided the decision against pursuing ECMO in this case.

This case illustrates the rapid deterioration seen in influenza-related ARDS when complicated by toxin-producing bacteria and reinforces the need for early, aggressive intervention. Radiographic signs of necrosis may be absent early in the disease course [6].

### 3.3. Necrotizing Pneumonia (NP) and Diagnostic Challenges

Our case reinforces the urgency of early dual-pathogen targeting, clinician awareness of influenza B severity, and the life-saving potential of vaccination. Future research should focus on biomarkers to identify high-risk patients and optimize adjunctive therapies for this lethal co-infection. A defining feature of this case was NP due to community-acquired MRSA, confirmed at autopsy. This severe complication involves lung tissue necrosis, microabscesses, and sometimes gangrene, with reported mortality up to 45% [6,23]. In children, *S. aureus* (particularly MRSA) and *Streptococcus pneumoniae* are leading causes [4]. Influenza B likely primed the lungs, and MRSA cytotoxins such as PVL induced leukocyte lysis and necrosis [10].

Histopathology showed microabscesses and thrombosed vessels, consistent with staphylococcal NP [6,24]. Clinical signs, including fever, leukopenia, and treatment failure, were present.

Although imaging did not show cavitation, this may reflect the limitations of plain radiography in early necrosis. CT imaging is the gold standard for diagnosing NP [6,25]. However, we could not perform it due to the patient’s critical instability.

### 3.4. Antibiotic and Antiviral Therapy

Guidelines recommend early empiric anti-MRSA therapy in severe influenza, without waiting for culture results [26]. Vancomycin alone may be inadequate; combination therapy with clindamycin or linezolid is preferred [26]. Our patient received vancomycin on admission and linezolid on Day 2 after MRSA confirmation. Given its anti-toxin effects, earlier use of linezolid might have been beneficial [16,26,27]. Moxifloxacin was later added as per the antibiogram and for potential additional anti-toxin coverage [26].

CDC and IDSA guidelines support continued initiation in influenza-positive critically ill patients [11,28], as late administration may still confer benefits in severe lower respiratory tract infections [12]. We initiated antiviral therapy on Day 8 of illness onset, which is beyond the recommended 48-hour window for maximal efficacy [11,29].

### 3.5. Hemodynamic Support and Adjunctive Therapies

In pediatric septic shock, norepinephrine is the recommended first-line vasopressor, while dopamine—associated with a higher risk of arrhythmias—may be used in refractory hypotension, particularly in multiorgan failure [1]. In our case, both agents were required and titrated to maximum doses to maintain perfusion.

Adjunctive therapies such as IVIG and cytokine hemoadsorption were employed for refractory shock. IVIG has been used in severe staphylococcal infections for its anti-toxin effects and may benefit cases of NP or toxic shock [30]. Despite aggressive treatment, the infection progressed; NP was confirmed at autopsy. Some of the literature supports early surgical consultation in suspected cases, though fulminant progression often precludes intervention [31,32]. Mortality in PVL-positive staphylococcal pneumonia can reach 56%, especially with shock and respiratory failure [6].

Cytokine hemoadsorption, used here due to elevated IL-6 and unrelenting shock, aims to reduce systemic inflammation. While pediatric data suggest potential hemodynamic improvement, evidence from randomized trials remains limited [33,34]. Ultimately, early antimicrobial therapy and organ support remain central to care.

### 3.6. Multiorgan Dysfunction Syndrome (MODS)

Sepsis-induced coagulopathy results from an overwhelming inflammatory response that activates the coagulation cascade [35]. In influenza and staphylococcal sepsis, cytokines such as IL-6 and TNF-α promote tissue factor expression and suppress anticoagulant pathways (e.g., protein C, antithrombin), leading to microthrombi and consumption of clotting factors [36]. Our patient developed DIC, managed with platelet and plasma transfusions. CRRT with cytokine hemoadsorption was initiated to mitigate hyperinflammation, though its efficacy in DIC is still uncertain [37]. Despite these interventions, coagulopathy persisted—consistent with the literature emphasizing that infection control remains the cornerstone of DIC reversal [38].

Sepsis-associated DIC nearly doubles mortality risk [39]. In this case, coagulopathy, high IL-6, and liver failure reflected the difficulty of reversing DIC in fulminant, toxin-driven sepsis.

Massive rhabdomyolysis (CK > 100,000 U/L), a rare but recognized complication of influenza B, was also observed [40]. Likely multifactorial, this was attributed to viral myositis, fever, hypoperfusion, and staphylococcal toxins [40]. The resulting pigment nephropathy likely worsened acute AKI, contributing to metabolic derangements such as hyperkalemia, acidosis, and inflammation [40]. CRRT with hemoadsorption was used to remove myoglobin and inflammatory mediators [41], but renal function deteriorated. Clinicians should monitor for rhabdomyolysis in severe influenza, which may signal impending MODS [42].

AKI developed early, with creatinine rising from 1.5 to 2.3 mg/dL despite hemodynamic support. Likely causes included septic shock-induced acute tubular necrosis (ATN), cytokine injury, and rhabdomyolysis [43]. Though MRSA can rarely cause glomerular damage, ischemic ATN is more typical in fulminant sepsis [44]. CRRT was initiated on Day 2 for worsening renal function, fluid overload, and acidosis. A cytokine adsorber was added to reduce systemic inflammation. While some studies suggest improved hemodynamics, a clear mortality benefit remains unproven [33,34]. In our case, despite CRRT, hemodynamic instability persisted, highlighting the severity of ARDS–AKI overlap, which significantly worsens prognosis [45].

By Day 4, the patient developed acute liver failure with transaminase elevation, INR > 2, and mild hyperbilirubinemia. This likely reflected ischemic hepatitis due to hypoperfusion and hypoxemia [46,47], compounded by inflammation and microthrombi as part of DIC [48]. Supportive care included perfusion optimization, avoidance of hepatotoxic drugs, and plasma transfusion. N-acetylcysteine was not administered, given the ischemic etiology. There is no specific therapy for sepsis-induced liver injury beyond supportive measures [49]. Early liver failure in septic shock is associated with high mortality [50].

The combination of ARDS, AKI, DIC, and liver failure indicated irreversible multi-organ dysfunction syndrome (MODS). A rising pSOFA score, from 11 to 15 by Day 5, captured this decline. In pediatric sepsis, a pSOFA ≥ 10 is associated with mortality rates up to 30% [51]. El-Mashad et al. found scores >6.5 predicted 30-day mortality with 80.9% sensitivity and 81.8% specificity [52].

Although IL-6 was the only cytokine tested, it was markedly elevated (>8350 pg/mL) and accompanied by elevated CRP, ferritin, D-dimer, and procalcitonin. This constellation supports a cytokine storm, which is commonly seen in severe toxin-mediated infections, such as PVL-associated MRSA [53]. Although PVL testing was unavailable, we can only speculate that the fulminant course combined with leukopenia, ARDS, and MODS is suggestive of a toxin-driven inflammatory response. High-volume plasma exchange has shown promise in select cases, but remains experimental and was not pursued here [49].

### 3.7. Preventive Measures and Recommendations

Initial outpatient management was suboptimal—azithromycin was prescribed, which does not cover influenza or MRSA. Empiric antibiotic use in viral illness can delay appropriate care and foster resistance [54]. Earlier influenza testing may have enabled timely antiviral therapy and referral, potentially altering the outcome. Unfortunately, by the time of admission, the disease had progressed to a fulminant state.

This case of fatal influenza B complicated by MRSA NP in a previously well adolescent is rare and underreported. Most of the existing literature focuses on influenza A (e.g., H1N1), making this influenza B case especially noteworthy [55]. Similar pediatric cases have reported rapid progression to ARDS, shock, and MODS within days of symptom onset [5,56]. Common features include leukopenia, coagulopathy, and poor outcomes despite intensive care, often associated with PVL-positive strains [5].

Though ECMO has been used as salvage therapy, outcomes remain mixed [22]. Such severe co-infections are more commonly described in adults, with only one similar fatal case reported in a Canadian teenager [57,58]. This case demonstrates a devastating sequence—MRSA sepsis, NP, ARDS, DIC, rhabdomyolysis, and MODS—rarely documented together. Despite guideline-based care, the synergy between influenza and MRSA proved fatal, emphasizing the need for early recognition and prevention, particularly through vaccination [59].

Clinicians should maintain a high index of suspicion for MRSA in pediatric influenza cases presenting with shock, leukopenia, or hemoptysis, as early targeted therapy can be lifesaving [26]. Vancomycin alone may be inadequate in influenza–MRSA pneumonia; in retrospect, initiating combination therapy (e.g., with clindamycin or linezolid) may have provided better toxin suppression. Absence of cavitation on imaging does not exclude necrosis—early CT should be considered when deterioration occurs despite therapy.

This case highlights the critical importance of annual influenza vaccination, even in healthy adolescents—a group in which vaccine uptake remains low across Europe, including Romania [7]. Despite being considered low-risk, unvaccinated youth can develop fulminant complications such as NP when influenza is followed by secondary bacterial infections. Influenza vaccination not only reduces primary viral illness but may also indirectly prevent life-threatening bacterial co-infections, particularly with PVL-positive *S. aureus*. Increasing vaccine coverage in this demographic is a vital, underutilized strategy for preventing severe outcomes like the one described here.

### 3.8. Study Limitations

This case report has several limitations. First, PVL testing was unavailable, so the diagnosis of toxin-mediated pathology was based on clinical features alone. Second, the patient’s instability precluded chest CT, which may have delayed the recognition of NP, as early CXR findings can be non-specific. Third, as a single case, generalizability is limited; however, it underscores the challenges of managing severe influenza–MRSA co-infection in resource-limited settings.

## 4. Conclusions

Based on this experience and the literature, we suggest the following: (1) consider MRSA co-infection early in severe influenza, especially with leukopenia or shock; (2) empiric dual-pathogen therapy should be considered in severe pediatric influenza presenting with shock or leukopenia, regardless of radiographic findings; (3) pursue early CT imaging if safe; and last but not least, (4) promoting influenza vaccination in adolescents could prevent catastrophic outcomes in adolescents without known comorbidities.

## Figures and Tables

**Table 1:** Full blood count and inflammatory marker data of the patient.

Reference Values	Day 1	Day 2	Day 3	Day 4	Day 5	Day 6
WBC (4–10.5 × 10^3^/uL)	1.73	1.67	1.65	20.09	38.17	45.12
ANC (1.8–8 × 10^3^/uL)	0.80	1.39	2.17	19.38	36.77	40.88
ALC (1.5–6.5 × 10^3^/uL)	0.69	0.11	0.24	0.6	1.06	1.71
RBC (4.2–5.6 × 10^6^/uL)	5.13	4.17	3.52	3.09	3.14	2.70
Hb (12.5–16.1 g/dL)	16	13.34	10.9	9.7	9.8	8.9
HCT (36–47%)	46.5	36.3	32.4	28.9	29.4	26.1
PLT (150–450 × 10^3^/uL)	56	20.8	13.9	29	28	63
CRP (0–0.5 mg/dL)	25.29	33.37	37.81	36.88	34.84	28.07
PCT (0–0.5 ng/mL)	675	1840	932	1210	904	349
IL 6 (0–17 pg/mL)	>5000	>8365	>8365	3657.24		
Ferritin (30–400 ng/mL)	980	1003.87	773.77	806.56		
ESR (2–12 mm/h)	4	16	30	70		25

(WBC—White blood cell; ANC—Absolute neutrophil count; ALC—Absolute lymphocyte count; RBC—Red blood cell; Hb—Hemoglobin; HCT—Hematocrit; PLT—Platelet count; CRP—C-reactive protein; PCT—Procalcitonin; IL-6—Interleukin-6; ESR—Erythrocyte sedimentation rate).

**Table 2:** Coagulation studies of the patient.

Reference Values	Day 1	Day 2	Day 3	Day 4	Day 5	Day 6
Fibrinogen (150–400)	238	303	401	696	744	329
D-dimer (0–0.55 mg/L)	17	17.30	16.67	10.60	5.08	
Prothrombin time (10.6–14 s)	18.1	23.7	21.8	12.8	11.5	25
INR (0.9–1.13)	1.61	2.18	1.98	1.09	0.97	2.31
Prothrombin concentration (70–130%)	45.6	32.3	71.2	95.7	106.9	30.2
APTT (21–40 s)	36.3	39.9	79	32.8	25.2	31.9

(INR—International Normalized Ratio; APTT—Activated partial thromboplastin time).

**Table 3:** Organ function and tissue injury studies of the patient.

Reference Values	Day 1	Day 2	Day 3	Day 4	Day 5	Day 6
Urea (11–45 mg/dL)	41.3	65.7	63.2	70.7	54.9	37.1
Creatinine (0.5–0.8 mg/dL)	1.50	2.30	2.46	2.80	2.35	2.27
eGFR mL/min/1.73 m^2^	49.01	32.32	33.34	29.88	33.39	32.39
Osmolarity (261–280 mosm/kg)	273.082				282.54	
Direct bilirubin (0–0.3 mg/dL)	0.56	0.96	1.51	1.68	1.55	
Indirect bilirubin (0–1.1 mg/dL)	0.17	0.18	0.22	0.21	0.15	
AST (17–59 U/L)	63	549	921	1350	1467	10,586
ALT (4–44 U/L)	27	78	175	240	268	2569
CK (55–370 U/L)	661	35,841	56,422	95,953	107,818	104,280
LDH (105–235 U/L)	631			3494	3245	

(eGFR—Estimated glomerular filtration rate; CK—Creatine kinase; LDH—Lactate dehydrogenase; AST—Aspartate transferase; ALT—Alanine aminotransferase).

## Data Availability

The original contributions presented in this study are included in the article. Further inquiries can be directed to the corresponding author.

## Abbreviations

The following abbreviations are used in this manuscript:

ALC	Absolute lymphocyte count
ALT	Alanine aminotransferase
ANC	Absolute neutrophil count
APTT	Activated partial thromboplastin time
ARDS	Acute respiratory distress syndrome
AST	Aspartate transferase
CK	Creatine kinase
CRP	C-reactive protein
CRRT	Continuous renal replacement therapy
CXR	Chest X-ray
DIC	Disseminated intravascular coagulation
DVT	Deep venous thrombosis
eGFR	Estimated glomerular filtration rate
ESR	Erythrocyte sedimentation rate
Hb	Hemoglobin
HCT	Hematocrit
ICU	Intensive care unit
IL-6	Interleukin-6
INR	International Normalized Ratio
IVIG	Intravenous immunoglobulin
LDH	Lactate dehydrogenase
MRSA	Methicillin-resistant Staphylococcus aureus
PCT	Procalcitonin
PEEP	Positive end-expiratory pressure
PICU	Pediatric intensive care unit
PLT	Platelet count
pSOFA	Pediatric Sequential Organ Failure Assessment
PT	Prothrombin time
PVL	Panton–Valentine leukocidin
RBC	Red blood cell
RT-PCR	Reverse transcription polymerase chain reaction
S aureus	Staphylococcus aureus
WBC	White blood cell
